# Dated tribe-wide whole chloroplast genome phylogeny indicates recurrent hybridizations within Triticeae

**DOI:** 10.1186/s12862-017-0989-9

**Published:** 2017-06-16

**Authors:** Nadine Bernhardt, Jonathan Brassac, Benjamin Kilian, Frank R. Blattner

**Affiliations:** 1 Leibniz Institute of Plant Genetics and Crop Plant Research (IPK), Gatersleben, Germany; 2 Present address: Crop Trust, Bonn, Germany; 3 German Centre for Integrative Biodiversity Research (iDiv) Halle-Jena-Leipzig, Leipzig, Germany

**Keywords:** Hybridization, Whole chloroplast genome, Phylogeny, Next-generation sequencing, Divergence times, Triticeae, Wheat, *Triticum*, *Aegilops*, *Psathyrostachys*

## Abstract

**Background:**

Triticeae, the tribe of wheat grasses, harbours the cereals barley, rye and wheat and their wild relatives. Although economically important, relationships within the tribe are still not understood. We analysed the phylogeny of chloroplast lineages among nearly all monogenomic Triticeae taxa and polyploid wheat species aiming at a deeper understanding of the tribe’s evolution. We used on- and off-target reads of a target-enrichment experiment followed by Illumina sequencing.

**Results:**

The read data was used to assemble the plastid locus *ndh*F for 194 individuals and the whole chloroplast genome for 183 individuals, representing 53 Triticeae species and 15 genera. We conducted Bayesian and multispecies coalescent analyses to infer relationships and estimate divergence times of the taxa. We present the most comprehensive dated Triticeae chloroplast phylogeny and review previous hypotheses in the framework of our results. Monophyly of Triticeae chloroplasts could not be confirmed, as either *Bromus* or *Psathyrostachys* captured a chloroplast from a lineage closely related to a *Bromus*-Triticeae ancestor. The most recent common ancestor of Triticeae occurred approximately between ten and 19 million years ago.

**Conclusions:**

The comparison of the chloroplast phylogeny with available nuclear data in several cases revealed incongruences indicating past hybridizations. Recent events of chloroplast capture were detected as individuals grouped apart from con-specific accessions in otherwise monopyhletic groups.

**Electronic supplementary material:**

The online version of this article (doi:10.1186/s12862-017-0989-9) contains supplementary material, which is available to authorized users.

## Background

The economically important grass tribe Triticeae Dumort. consists of approximately 360 species and several subspecies in 20-30 genera. Triticeae taxa occur in temperate and dry regions of the World and harbour the important cereals bread wheat (*Triticum aestivum*), barley (*Hordeum vulgare*), rye (*Secale cereale*) and their wild relatives [1, 2]. Yet there is no good understanding of the relationships among Triticeae taxa, although a multitude of molecular phylogenies have been produced [3–11]. The acceptance levels of taxa vary greatly among authors on the genus-level and below (for recent reviews see [1, 12, 13]). One important reason is the complex mode of evolution within Triticeae. The majority of species are allopolyploids and many of them likely have originated repeatedly, involving genetically different parent species [14–19]. Bread wheat is the most prominent polyploid and evolved via consecutive hybridizations of three diploids and thereby combines three related genomes (named **A**, **B** and **D**) [7, 20]. In Triticeae and many other crops such genomes were defined through cytogenetic characterization of chromosomes together with the analysis of their pairing behaviour in interspecific and intergeneric crosses (for reviews see [1, 12, 21]). It is assumed that diploid species and monogenomic taxa are the basic units within Triticeae and that the heterogenomic polyploids form a second level of taxonomic entities [22, 23].

Triticeae are known to have low barriers against hybridization, which result in mixed or even recombinant phylogenetic signals from nuclear data [10, 20]. In contrast, phylogenetic analyses of plastid sequences provide clear information on maternal lineages, as organelles are mostly uniparentally inherited and non-recombining in angiosperms [24], although chloroplast capture [25] can result in deviating phylogenetic hypotheses. Yet, plastid sequence data is limited for Triticeae. Studies based on a tribe-wide taxon sampling are rare and focused on single or few plastid markers [9, 26–28]. To date, the number of whole plastid genome sequences is increasing [29–34], however, entire chloroplast genomes are mainly available for the domesticated taxa and their closest relatives. These previous studies provide only limited insight in the maternal phylogeny of Triticeae, as only one to few accessions per taxon were included and often support values for the taxonomic units are low [26, 28, 35].

Here we present phylogenetic analyses of chloroplast sequences based on a comprehensive set of monogenomic Triticeae species plus allopolyploid representatives of the wheat group (i.e. taxa belonging to the genera *Aegilops* and *Triticum*). For each species we included multiple individuals to sample part of the intraspecific variation. We performed a target-enrichment and next-generation sequencing (NGS) approach that, among nuclear loci (which will be published elsewhere), targeted the chloroplast *ndh*F gene. Since chloroplasts occur in high copy number in the plant cell, they represent a large fraction of the off-target reads when sequencing reduced complexity libraries, which can be used to assemble almost complete chloroplast genomes [36]. Our dataset was complemented by chloroplast genomes stored in the GenBank database. Multispecies coalescent (MSC) analyses based on *trn*K-*mat*K, *rbc*L and *ndh*F were used for dating of the major splits within the evolution of the tribe and to reconsider the monophyly of the Triticeae chloroplast lineages.

## Methods

### Plant materials

Aiming at a good representation of taxa for phylogenetic inference we analysed 194 individuals representing approximately 53 species belonging to 15 genera (depending on taxonomic treatment applied) of the grass tribe Triticeae and included *Bromus* and *Brachypodium* accessions as outgroup taxa (Table 1, Additional file 1: Table S1). The accessions were acquired from the International Center for Agricultural Research in the Dry Areas (ICARDA), the seed bank of the Leibniz Institute of Plant Genetics and Crop Research (IPK), the National Small Grain Collection of the US Department of Agriculture (USDA), the Czech Crop Research Institute, and the Laboratory of Plant Genetics (Kyoto University). Additional seed material was collected during field trips. Multiple accessions per species and intra-specific entities were selected if possible. All materials were grown from seed and identified based on morphological characters if an inflorescence was produced. Plant material obtained from germplasm repositories that was found to be in conflict with its taxonomic affiliation was only included in the analyses if the taxon could be unequivocally determined. Vouchers of the morphologically identified materials (Additional file 1: Table S1) were deposited in the herbarium of IPK (GAT).

**Table 1:** Overview of Triticeae and outgroup taxa considered

Species	Genome	Ploidy (N)	Distribution area
*Aegilops bicornis* Jaub. & Spach	S*	2× (4)	SE Mediterranean
*Aegilops biuncialis* Vis.	UM	4× (4)	SW-SE Europe, N Africa, SW Asia
*Aegilops columnaris* Zhuk.	UM	4× (2)	SW Asia
*Aegilops comosa* Sm.	M	2× (4)	Balkans
*Aegilops crassa* Boiss.	DM/DDM	4× (1)/6× (2)	SW Asia
*Aegilops cylindrica* Host	DC	4× (2)	SE Europe, W Asia
*Aegilops geniculata* Roth	MU	4× (3)	E Europe, W Asia, Macaronesia
*Aegilops juvenalis* Eig	DMU	6× (2)	SW Asia,
*Aegilops kotschyi* Boiss.	S*U	4× (1)	SW Asia, NE Africa
*Aegilops longissima* Schweinf. & Muschl.	S*	2× (5)	E Mediterranean
*Aegilops markgrafii* (Greuter) K. Hammer	C	2× (5)	NE Mediterranean
*Aegilops neglecta* Req. ex Bertol.	UM/UMN	4× (2)/6× (2)	Mediterranean to SW Asia
*Aegilops peregrina* (Hack.) Maire & Weiller	SU	4× (1)	SW Asia, N Africa
*Aegilops searsii* Feldman & Kislev	S*	2× (5)	E Mediterranean
*Aegilops sharonensis* Eig	S*	2× (1)	Israel, Lebanon
*Aegilops speltoides* Tausch	S	2× (6)	E Mediterranean
*Aegilops tauschii* Coss.	D	2× (4)	SW–C Asia
*Aegilops triuncialis* L.	UC	4× (2)	Mediterranean to SW Asia
*Aegilops umbellulata* Zhuk.	U	2× (3)	SE Europe, SW Asia
*Aegilops uniaristata* Vis.	N	2× (3)	SE Europe
*Aegilops ventricosa* Tausch	DN	4× (2)	SW Europe, N Africa
*Agropyron cristatum* (L.) Gaertn.	P	2× (2)/4× (4)	S Europe, NECW Asia
*Amblyopyrum muticum* (Boiss.) Eig	T	2× (6)	Turkey
*Australopyrum retrofractum* (Vickery) A. Löve	W	2× (4)	SE Australia
*Dasypyrum villosum* (L.) P. Candargy	V	2× (5)	SW–SE Europe, Caucasus
*Eremopyrum bonaepartis* (Spreng.) Nevski	Ft/Xe/FtXe	2×/4× (5)	SE–E Europe, WC Asia
*Eremopyrum triticeum* (Gaertn.) Nevski	Ft	2× (3)	SE–E Europe, WC Asia
*Henrardia persica* (Boiss.) C.E. Hubb.	O	2× (4)	SE Europe, SW Asia
*Heteranthelium piliferum* Hochst. ex Jaub. & Spach	Q	2× (4)	SE Europe, SW Asia
*Hordeum bulbosum* L.	I	4× (1)	Mediterranean to C Asia
*Hordeum marinum* Huds.	Xa	2× (1)	Mediterranean
*Hordeum murinum* L.	Xu	2× (1)	Mediterranean to C Asia
*Hordeum pubiflorum* Hook. f.	I	2× (1)	S Argentina
*Hordeum vulgare* L.	H	2× (2)	SW Asia
*Psathyrostachys juncea* (Fisch.) Nevski	Ns	2× (6)	E Europe, NC Asia
*Pseudoroegneria cognata* (Hack.) A. Löve	St	6× (1)	SW Asia, West Himalaya
*Pseudoroegneria spicata* (Pursh) A. Löve	St	2× (2)/6× (1)	NW of Northern America
*Pseudoroegneria stipifolia* (Czern. ex Nevski) A. Löve	St	2× (1)/4×(2)	E Europe, N Caucasus
*Pseudoroegneria strigosa* (M. Bieb.) A. Löve	St	2× (2)/6× (2)	Balkans, Crimea
*Pseudoroegneria tauri* (Boiss. & Balansa) A. Löve	St	2× (5)	E Mediterranean, S Caucasus
*Secale cereale* L.	R	2× (4)	Turkey
*Secale strictum* C. Presl	R	2× (4)	S Europe, SW Asia, N Africa
*Taeniatherum caput-medusae* (L.) Nevski	Ta	2× (6)	S Europe, SW Asia, N Africa
*Thinopyrum distichum* (Thunb.) A. Löve	E	4× (2)	S Africa
*Thinopyrum* spp*.* Löve	E	6× (1)/8× (2)	SE Europe, SW Asia, N Africa
*Triticum aestivum* L.	BAD	6× (6)	Caucasus, Iran
*Triticum monococcum* L.	A	2× (10)	Turkey
*Triticum timopheevii* (Zhuk.) Zhuk.	GA	4× (7)	SW Asia
*Triticum turgidum* L.	BA	4× (10)	Lebanon
*Triticum urartu* Thumanjan ex Gandilyan	A	2× (5)	E Mediterranean, Caucasus
*Triticum zhukovskyyi* Menabde & Ericzjan	GAA	6× (1)	Caucasus
*Brachypodium distachyon* (L.) P. Beauv.		4× (1)	S Europe, SW Asia, N Africa
*Brachypodium pinnatum* L.) P. Beauv.		4× (1)	Europe, NCW Asia, NE Africa
*Bromus inermis* Leyss.		4× (1)	SW Asia, Caucasus
*Bromus tectorum* L.		4× (1)	Europe, SW Asia, N Africa

The genome, determined ploidy levels, number of included accessions (N), and the main native distribution for all taxa sequenced in this study is given. The genomes names of allopolyploid *Aegilops* taxa are follwing Kilian et al. [74] and Li et al. [84] ﻿for S*. Genome denominations for *Hordeum* follow Blattner [107], and Bernhardt [12] for the remaining taxa. Different seed banks adopt different taxonomic treatments that may vary in the number of (sub)species recognized. More comprehensive information about the used accessions, including the species names used in the donor seed bank and the country of origin is provided in Additional file 1: Table S1

### Laboratory work

Flow-cytometric measurements were conducted to determine the ploidy level for all accessions. All analyses followed the protocol of Doležel et al. [37] on a CyFlow Space flow cytometer (Partec). At least 7500 nuclei were counted. Only measurements with a coefficient of variation (CV) for sample and standard peak <4% were accepted. Samples that recurrently produced CV values >4% were repeated in Galbraith’s buffer containing 1% polyvinylpyrrolidon (vol/vol) and 0.1% Triton X-100 (vol/vol). At least three measurements per species were carried out. If only a single accession of a species could be retrieved from a seed bank, its ploidy level was estimated three times. Samples of the same species were processed on at least two different days to account for instrument drifts.

Genomic DNA was extracted either from 10 mg silica-dried leaves using the DNeasy Plant Mini Kit (Qiagen) or from 5 g of freeze-dried leaves using the cetyltrimethyl-ammonium bromide (CTAB) method [38]. DNA quantifications were done using the Qubit dsDNA BR Assay (Life Technologies) or the Quant-iT PicoGreen dsDNA Assay Kit (Invitrogen) on a Tecan Infinite 200 microplate reader according to the manufactures instructions. The LE220 Focused-Ultrasonicator (Covaris) was used to shear 3 μg genomic DNA in 130 μl TE buffer for every sample into fragments having an average length of 400 bp with the following settings: instantaneous ultrasonic power (PIP) 450 W, duty factor (df) 30%, cycles per burst (cpb) 200. The treatment was applied for 100 s. The sheared DNA was used in a sequence-capture approach (SureSelect^XT^ Target Enrichment for Illumina Paired-End Sequencing, Agilent Technologies) targeting at 450 nuclear single-copy loci aiming for 0.01–0.02% of a Triticeae genome. Baits complementary to chloroplast *ndh*F based on 628 bp of available *Hordeum vulgare, Aegilops tauschii*, *Pseudoroegneria spicata, Triticum urartu* (identical to EF115541.1, JQ754651.1, KJ174105.1 and AF056180.1, respectively) and 2073 bp of *Brachypodium distachyon* (identical to AF251451.1) sequences were designed as well. The pairwise sequence identity was larger then 99% among Triticeae taxa and 96% when comparing the Triticeae taxa with *Brachypodium*. Baits were designed to cover the entire 2073 bp of *ndh*F as well as each polymorphism between the reference sequences at least five times. After the enrichment procedure all samples were barcoded and pooled (following [39, 40]) at equimolar ratios. Capture libraries were sequenced on the Illumina HiSeq 2000 or MiSeq. The flowcells were loaded aiming for a sequencing coverage of at least 40X.

### Data assembly

We used the captured *ndh*F and the off-target read fraction (i.e. reads for which no capture probes were designed in the target-enrichment experiment) to assemble whole chloroplast genomes. Geneious versions R8–R10 (Biomatters Ltd.) were used for quality control and downstream analyses. Read pairs were set with an average insert size of 300 bp and bases with an error probability above 5% were trimmed. Chloroplast genomes were assembled in a two-step procedure consisting of (1) the generating of a species-specific reference sequences followed by (2) the creation of individual-based chloroplast assemblies.

In the first step we assembled species-specific chloroplast sequences by combining reads of multiple accessions of a single species. This increased the coverage for a species-specific chloroplast genome compared to the usage of data of an individual sample only. In a few cases single accessions were found to contribute a large amount of variation to these assemblies. These accessions were excluded from species-specific assemblies (Additional file 1: Table S1). The reads were either mapped to GenBank sequences of conspecific or closely related taxa (for *Aegilops*, *Hordeum* and *Triticum* species), or to *Hordeum vulgare* (EF115541), a well-studied basal organism in Triticeae, for taxa lacking conspecific chloroplast genomes in GenBank. One inverted repeat was cleaved off the GenBank sequences as no sequence variation has been found between the inverted repeats of the same chloroplast genome. A careful comparison of Triticeae chloroplast genomes available in GenBank showed a large amount of insertions and deletions (indels) among the sequences from single species. In case several chloroplast genomes per species were retrieved from GenBank, those were aligned and an annotated consensus was created as reference to check for intraspecific indels. Then a stringent read mapping approach was used that considered only reads with mates mapping in proper distance according to the insert size (±50%). This was done to avoid the inclusion of chimerical Illumina reads, which have been reported to occur frequently [41]. All read mappings were performed using the Geneious mapper with five iterations, allowing at maximum 15% of mismatches per read and a maximum gap size of 1000 bp to encompass large deletions. The assembly results were compared and manually checked for inconsistencies (i.e. indels the assembler was unable to resolve). Consensus sequences were called using the 50% majority rule. Up to five rounds of mapping and inspection were performed, each time using the contig obtained previously.

In the second step, for each sequenced individual chloroplast sequences were assembled by mapping all reads to their species-specific consensus sequence generated in step (1). Read mappings were performed using the Geneious mapper with five iterations, allowing at maximum 10% of mismatches per read and a maximum gap size of 100 bp. The assembly results were manually checked for inconsistencies. No global coverage threshold was applied as the read coverage for single accessions were relatively low. However, single nucleotide polymorphisms (SNPs) compared to the reference covered by a single read were masked. Finally consensus sequences were called using the ‘Highest Quality’ option, which is able to resolve conflicts between reads because it takes the relative residue quality into account. ‘N’ were called for positions without coverage. Whole chloroplast sequences with more than 50% missing data were excluded from further analyses.

A multiple sequence alignment of the whole chloroplast genomes generated in step (2) plus a set of GenBank-derived sequences (Additional file 1: Table S1) was generated using Mafft 7 (http://mafft.cbrc.jp/alignment/software; accessed in November 2016; [42]) applying the auto algorithm in combination with the ‘nwildcard’ option. The alignment was manually curated. The sequences generated in the scope of this study were annotated by comparing them to the annotations of the GenBank accession number KJ592713 [43] in Geneious. All sequences were submitted to GenBank (accession numbers KX591961-KX592154 and KY635999-KY636181). The number of parsimony-informative positions was inferred using PAUP* 4.0b10 [44].

### Phylogenetic analyses

We performed a Bayesian phylogenetic analysis for *ndh*F, as the sequence of this locus could be retrieved for all individuals without any missing data. First, unique *ndh*F haplotypes were identified using TCS 1.2.1 [45]. The best-suited model of sequence evolution was identified on the data matrix of unique haplotypes with jModelTest 2.1.4 [46] using the default parameters. The Bayesian information criterion (BIC; [47]) was selected for model choice because of its high accuracy [46] and its tendency to favour simpler models than the Akaike information criterion (AIC; [48]). Bayesian inference (BI) was performed in MrBayes 3.2.6 [49] using the model inferred by jModelTest. BI consisted of four independent analyses each running for 20 million generations and sampling a tree every 1000 generations.

BI of the whole chloroplast genome alignment were run with MrBayes 3.2.6 on the CIPRES (Cyberinfrastructure for Phylogenetic Research) Science Gateway 3.3 [50] for two datasets: (1) the complete alignment and (2) one alignment with positions having more than 50% of missing data being masked in Geneious version R10. The best-fitting models of sequence evolution were estimated by making the Monte Carlo Markov chain (MCMC) sampling across all substitution models ([51]; ‘lset nst = mixed’). For each dataset we performed three analyses, testing the impact of different rate settings, i.e. (1) a gamma-distributed rate variation, (2) a proportion of invariable sites and (3) with both combined to be able to identify the best-suited substitution model by comparing the posterior probabilities with AIC through MCMC (AICM; [52]), which is less computing intensive though not as accurate as the application of Bayes factors [53], in Tracer. Each analysis was performed with two independent Metropolis coupled MCMC analyses each with four sequentially heated chains (temperature set to 0.05) until the standard deviation of split frequencies reached 0.009, a maximum of 10 million generations or the maximum runtime of CIPRES. Trees were sampled every 500 generations. For all Bayesian analyses conducted *Brachypodium distachyon* (EU325680) was set as outgroup and the convergence of the runs was assessed in Tracer v. 1.6 [54]. A consensus tree was computed after deleting a burn-in of the first 25% of trees.

Additionally, a Bayes factor (BF; [55]) analysis was carried out for the *ndh*F dataset to further evaluate the monophyly of Triticeae chloroplasts. Mean marginal log-likelihoods were computed using the stepping-stone sampling [56] in MrBayes 3.2.6 [49] for monophyletic and polyphyletic relationships of Triticeae and the substitution model as identified in jModelTest. Each analysis consisted of two million generations with four independent runs of four parallel chains. The BF was evaluated using ten as a cut-off value [57].

### Estimating divergence times using *trn*K-*mat*K, *rbc*L and *ndh*F

We inferred a calibrated phylogeny for the three plastid loci *trn*K-*mat*K, *rbc*L and *ndh*F. First, we tested the robustness of the calibration of the most recent common ancestor (MRCA) of *Brachypodium* and Triticeae when increasing the sampling for Triticeae from 12 to 37 species compared to Marcussen et al. [20]. For this a Bayesian coalescence analysis based on *trn*K-*mat*K, *rbc*L and *ndh*F for the subfamily Pooideae was performed. The same GenBank sequences were assembled to form a contiguous sequence as described and used in Marcussen et al. [20]. This set of GenBank accessions was complemented with sequences assembled in this study whenever additional taxa or more sequence information from a certain taxon could be added. Following Marcussen et al. [20] we restricted ourselves to one sequence per species. We used the species-specific sequences from step (1) of the sequence assembly procedure, over the selection of a single accession per taxon, comparable to Pelser et al. [58]. This allowed us to employ all phylogenetic information available for a taxon and to overcome stretches of missing data. Conspecific sequences used for consensus inference showed 99.96 – 100% of identical sites. The best partitioning schemes and DNA substitutions models were inferred with PartitionFinder [59, 60] comparing all possible partitioning schemes. The analysis was carried out using the combination of age priors for analyses 2, 4, 6, 10 and 17 as published in Marcussen et al. [20] in Beast 2.4.1 [61]. For each setting one replicate was performed. Priors on the root age were estimated as stem node (‘use originate’). We found the divergence time of *Brachypodium* and Triticeae as inferred by Marcussen et al. [20] to be robust. Second, we performed a multispecies coalescent (MSC) analysis using it as the secondary calibration point in million years ago (Ma) as normally distributed priors for the root of *Brachypodium*-Triticeae (mean 44.44 Ma ± 3.53) on *trn*K-*mat*K, *rbc*L and *ndh*F for all Triticeae accessions. We excluded gene sequences of *trn*K-*mat*K and *rbc*L if they showed more than 50% of missing data and sequences of all polyploid wheat accessions. Sequences of *Zea mays*, *Oryza sativa*, *Brachypodium distachyon* and two *Bromus* species were included as outgroup taxa. The taxa *Triticum monococcum* and *T. boeoticum*, *Secale cereale* and *S. vavilovii*, *Pseudoroegneria tauri* and *Ps*. *libanotica*, *Taeniatherum caput-medusae* and *Tae*. *crinitum*, *Agropyron cristatum* and *Agr. cimmericum* were each subsumed under the same species name (Additional file 1: Table S1), as no pronounced genetic variation were detected in the analysis of whole chloroplast sequences. Hereby we were following existing taxonomic treatments, which already unify these taxa under a single species name (see, e.g. [62]).

We performed MSC analyses for a dataset including *Psathyrostachys* and another one without it to evaluate the impact of this taxon on divergence times. Monophyly of Triticeae was not enforced for either analysis as suggested by the Bayes Factor analysis. For each dataset, first, the best partitioning schemes and DNA substitution models were inferred with PartitionFinder searching all partitioning schemes. The analysis was run with the substitution models being linked, the Yule species tree prior, as well as the piecewise linear and constant root population model. Since the rate constancy was systematically rejected for all loci by the likelihood-ratio test [63], an uncorrelated lognormal clock model ([64]; uniform ucld.mean: min 0, max 0.01) was used. Trees were sampled every 5000 generations. Four independent analyses were performed and each was run for 600 million generations. All MSC analyses were run using the Beagle library [65]. Effective sample sizes (ESS) and convergence of the analyses were assessed using Tracer v. 1.6 [54]. An appropriate burn-in was estimated from each trace file, and the four analyses were combined with LogCombiner as part of the Beast package. Then a maximum clade credibility (MCC) tree was summarised with TreeAnnotator and visualized with FigTree 1.4.2 [66].

## Results

### Ploidy levels

Flow cytometric measurements were performed for all accessions to be able to distinguish between different ploidy levels for accessions of the same species (Additional file 1: Table S1). We identified di- and tetraploid accession for *Agropyron cristatum, Eremopyrum bonaepartis, Pseudoroegneria stipifolia and Ps. strigosa*, and detected tetra- and hexaploid cytotypes for *Aegilops crassa* and *Ae. neglecta*. Flow cytometric measurements were used as additional information to confirm species affiliations [67]. For example, comparing of the genome sizes measured for the diploid species *Thinopyrum bessarabicum* and *Th. elongatum* to the data from the Kew Angiosperm DNA C-values database revealed that the analysed accessions actually represent polyploids instead of diploids. For more information on problematic material from seed banks see Additional file 1: Table S1.

### Sequence assembly

The target-enrichment protocol and Illumina sequencing were applied to 194 accessions, covering 53 species of 15 genera (dependent on the applied classificatory system) and three outgroup species (i.e. *Bromus* and *Brachypodium*, Table 1, Additional file 1: Table S1). Whole chloroplast genomes were assembled in a two-step procedure via (1) an intermediate step of generating a species-specific reference if there was none available in GenBank and (2) the assembly of the chloroplast of each accession via read mapping to sequences from step (1).

The average coverage of the chloroplast genome varies largely between single samples and depends mainly on the actual sequencing depth. Between approximately 50% and 90% of the reads mapping to the chloroplast mapped to *ndh*F (Additional file 2: Table S2), which was included in the bait design. Thus, the *ndh*F gene could be assembled for all accession without missing data. We identified 64 unique haplotypes when comparing the *ndh*F gene data plus the sequences downloaded from GenBank (Additional file 1: Table S1). The alignment of these 64 haplotypes had a total length of 2232 bp with 186 (8.3%) parsimony-informative sites.

The entire alignment of whole chloroplast genome sequences comprised 222 sequences, 39 of them were downloaded from GenBank. This alignment ranged from *psb*A in the large single-copy region to partial *ndh*H in the small single-copy region and had a total length of 123,531 bp. It had 9064 (7.3%) parsimony-informative positions. The data matrix included 9.3% of missing data (‘N’). These randomly distributed stretches of missing data occur in the alignment in regions where the sequencing coverage was insufficient. Additionally the matrix revealed 7.5% of gaps due to length variation between taxa. In several cases taxa showed long indels in intergenic regions, thus the same 900 bp deletion was found between *rpl*23 and *ndh*B in *Pseudoroegneria*, *Thinopyrum* and *Dasypyrum.* Many short indels (3–40 bp) were found in introns of coding genes (e.g. *ysf3*) and intergenic spacers. A variant of this alignment, having regions with 50% of missing data being removed, had a total length of 114,788 bp. In this case 8717 (7.6%) positions of the alignment were parsimony informative, while 9.2% of the characters were constituted by N’s and 0.8% of gaps. Alignment masking mainly excluded regions of length variation due to short repeat motives in intergenic regions. With only few substitutions per chloroplast intraspecific variation was generally very low.

The alignment revealed insertions unique to some GenBank sequences whose true occurrence could not be confirmed by our data: no reads from our analysed individuals mapped to these insertions. Moreover, BLAST searches of these regions returned mitochondrial and/or nuclear genomic data as best hit (e.g. KC912690, KC912692, KC912693, KC912694) indicating assembly artefacts. Those GenBank sequences were excluded from further analyses (Additional file 1: Table S1).

### Phylogenetic analyses

**Fig. 1:**
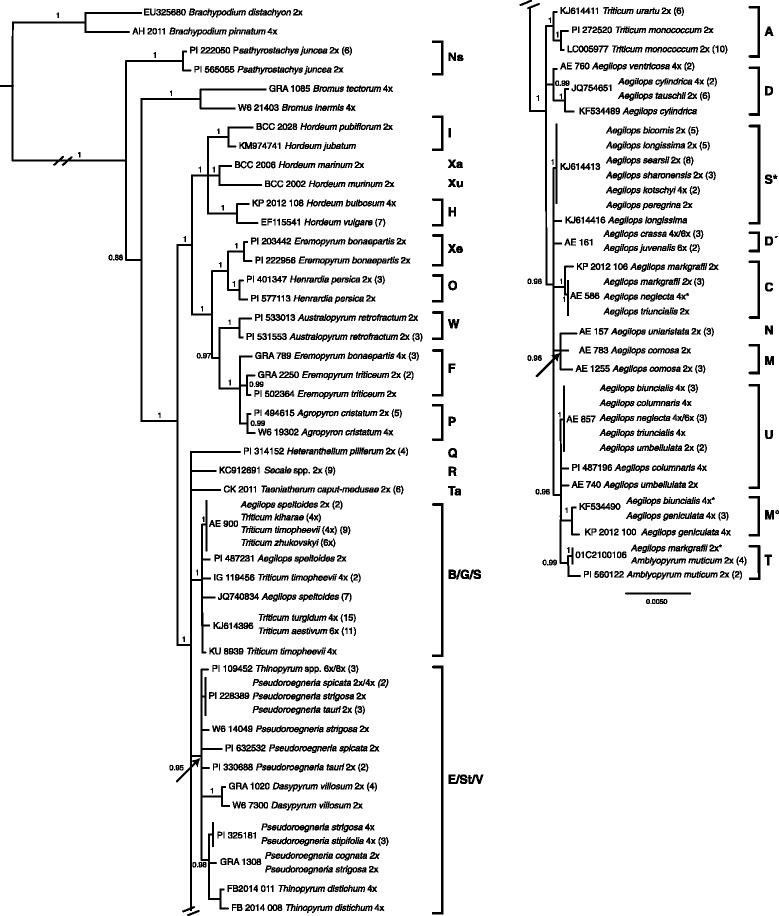
Phylogenetic tree derived from 2232 bp of the chloroplast locus *ndh*F and Bayesian inference. The multiple sequence alignment consisted of 64 unique haplotypes that originated from 194 accessions sequenced in the scope of this study and 41 sequences retrieved from GenBank. *Brachypodium distachyon* was set as outgroup taxon. Posterior probabilities (pp) for the main clades are depicted next to the nodes if they were higher then 0.75. Each unique haplotype is named with a distinctive identifier. For detailed information which accession possesses which haplotype and species synonyms see Additional file 1: Table S1. The ploidy level is indicated behind taxon labels. If there are multiple accessions per taxon sharing the same haplotype, the number of accessions is provided behind the taxon label. Single accessions grouping apart from other accessions of their taxon are marked with an *asterisk*. To the right the genomic groups are shown*. Arrows* with support values indicate the nodes they refer to

We performed a BI analysis on the set of 64 unique *ndh*F haplotypes with the model of sequences substitution set to GTR + G [68, 69], as identified by jModelTest. The phylogenetic tree obtained from *ndh*F (Fig. 1) shows Triticeae to be paraphyletic, as the lineage of *Psathyrostachys* appears to have diverged before the lineage of *Bromus*, although the position of *Bromus* is with a posterior probability (pp) of 0.88 not well supported. The branch lengths for the *Bromus* group are considerably longer compared to *Psathyrostachys*. The topology shows that individuals of most species and/or genera form monophyletic groups. However, *Eremopyrum bonaepartis* is polyphyletic, as the diploid plastid type of *E. bonaepartis* groups as sister to *Henrardia persica*, while the haplotypes of all tetraploid *E. bonaepartis* and diploid *E. triticeum* form a clade with *Agropyron cristatum*. A common maternal ancestor can be hypothesized for *Agropyron*, *Australopyrum*, *Eremopyrum* and *Henrardia* as these taxa form a well supported clade, which is sister to the clade of *Hordeum* species. The clades formed by the genera *Heteranthelium*, *Secale* and *Taeniatherum* are placed on a polytomy together with a clade formed by taxa having a **B**, **G** or **S** genome [i.e. *Aegilops speltoides* (**S**) and all polyploid *Triticum* taxa (**B**/**G**)], the clade of taxa with an **E**, **St** or **V** genome (i.e. *Thinopyrum*, *Pseudoroegneria* and *Dasypyrum*), and the clade of all remaining *Aegilops*, *Amblyopyrum* and diploid *Triticum* taxa. *Pseudoroegneria* appears paraphyletic, as *Dasypyrum* and *Thinopyrum* haplotypes group within this clade. The backbone of this clade represents a polytomy. Notably the placement of the otherwise monophyletic *Dasypyrum* is not supported. Several different haplotypes can be distinguished for various species of *Pseudoroegneria* itself (e.g. *Ps. spicata*, *Ps. strigosa*, *Ps. tauri*, *Ps. stipifolia*). Furthermore, the two **A**-genome species *Triticum urartu* and *T. monococcum* are monophyletic. Also all **D**-genome species (i.e. *Ae. tauschii*, *Ae. cylindrica* and *Ae. ventricosa*) form a clade. Both genomic groups are located on a polytomy together with the remaining *Aegilops* species and *Amblyopyrum*. *Aegilops crassa* and *Ae. juvenalis* (**D’**) group apart from the other **D** taxa and show a *ndh*F haplotype with less nucleotide differences to S* than to **D** chloroplast lineages (i.e. one SNP difference to S* vs. three and five SNPs to **D**). All diploid and polyploid S* species sequenced in the scope of this study share the same *ndh*F haplotype. *Aegilops comosa* (**M**) and *Ae. uniaristata* (**N**) are sister species. All **U**-genome taxa fall into the same clade together with *Aegilops geniculata* (**M°**) and *Amblyopyrum muticum* (**T**). *Aegilops triuncialis* accessions possess **U** as well as **C** haplotypes.

Sometimes, single accessions of a species group within the otherwise monophyletic clade of another species. Thus, the accession AE_1831 of *Aegilops markgrafii* (**C**) falls into the clade of *Amblyopyrum muticum* (**T**) while KP_2012_119 of *Aegilops biuncialis* (**U**) falls within *Ae. geniculata* (**M°**). The accession AE_586 of *Aegilops neglecta* (**U**) groups together with *Ae. markgrafii* (**C**). Further, intraspecific variation within *ndh*F was found in several cases, for example, for *Aegilops comosa*, *Ae. speltoides, Amblyopyrum muticum*, and *Dasypyrum villosum.* With a score of 36.4, BF strongly favours Triticeae chloroplasts as paraphyletic (Additional file 3: Table S3) when *Psathyrostachys* is included in the analysis.

**Fig. 2:**
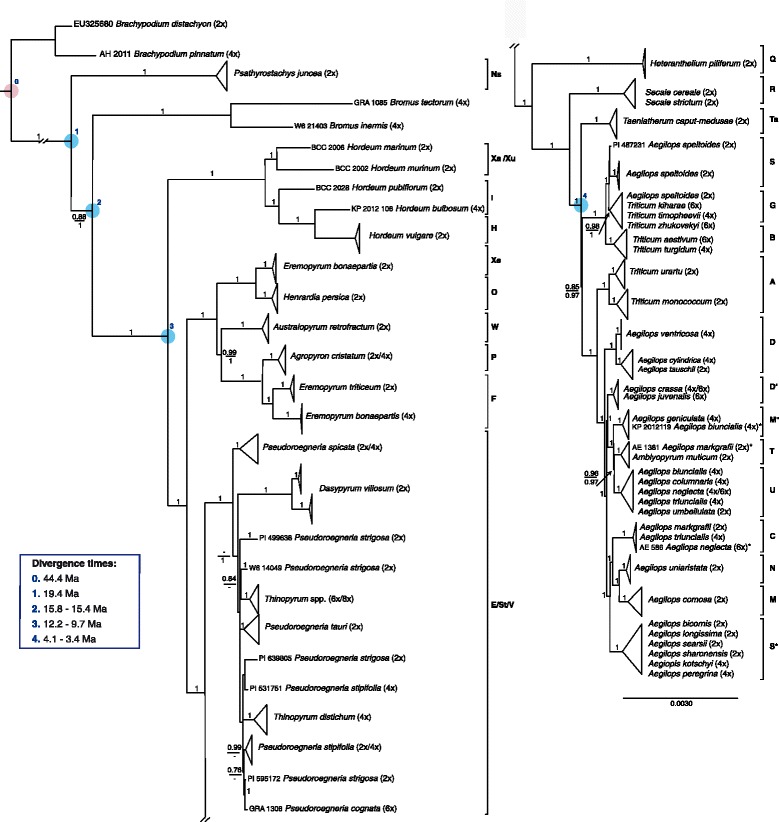
Phylogenetic tree derived from an alignment of whole genome chloroplast sequences via Bayesian phylogenetic inference. The multiple sequence alignment comprised 183 genomes assembled in the present study and 39 genomes that were downloaded from GenBank. *Brachypodium distachyon* was defined as outgroup taxon. The tree shown corresponds to an analysis based on the complete alignment of 123,531 base pairs (bp). Clades were collapsed into triangles to reflect the main groupings. The area of the *triangles* reflects the genetic variation contained in a certain clade. Posterior probabilities (pp) for the main clades are depicted next to the nodes if they were higher then 0.75. Support values of a second Bayesian phylogenetic analysis based on 114,788 bp of whole chloroplast genomes, where alignment positions with more than 50% of missing data were masked, are shown below the values of the corresponding nodes in the complete chloroplast analysis if the values differed between analyses. Ploidy levels are provided in brackets after the taxon labels. Single accessions grouping apart from other accessions of their taxon are highlighted with an *asterisk*. To the *right* the genomic groups are indicated. The *red circle* represents the secondary calibration point from Marcussen et al. [20] used for node calibrations in multispecies coalescent analyses (MSC). Major nodes are shown in *blue* and their estimated ages in million years are given in the *box*. Two age values for the same node correspond to the analysis with (first value) and without the inclusion of *Psathyrostachys* (second value). For more information on the results of the MSC analyses see Additional file 5: Figure S2 and Additional file 6: Figure S3. For the full representation of the tree showing the grouping of all single accessions see Additional file 4: Figure S1. For species synonyms see Additional file 1: Table S1. Arrows with support values indicate the nodes they refer to

As the resolution of the phylogenetic tree from the *ndh*F dataset is not sufficient to distinguish between more recently diverged taxa, the whole chloroplast genome dataset was phylogenetically analysed by BI using an alignment of the entire chloroplast genomes and a variant of it were positions having more then 50% of missing data have been masked. In both cases MrBayes revealed a *gtrsubmodel* in combination with gamma-distributed rate variations as best-suited substitution model. The topologies (Fig. 2, Additional file 4: Figure S1) returned from both analyses are mainly congruent to each other and to the *ndh*F tree. However, nodes of deep splits supported moderately for the complete plastid data matrix show higher support in the dataset where low-covered regions have been masked. This is, for example, the case for the split of the ancestor of *Bromus.* The branch length differences between *Bromus* and *Psathyrostachys* are in agreement with the *ndh*F tree. In contrast to the *ndh*F dataset, the whole chloroplast phylogenies are able to provide a hypothesis of the relationships between all major genomic groups. They suggest that the **E**, **St**, and **V** clade (i.e. *Thinopyrum*, *Pseudoroegneria* and *Dasypyrum*) diverged before *Heteranthelium*, which in turn split before *Secale* and *Taeniatherum. Pseudoroegneria spicata* forms its own clade that diverged first from *Dasypyrum* and the remaining taxa within this clade. However, the *Dasypyrum* chloroplast genomes are characterized by rather long branches compared to other taxa in this clade. Furthermore, *Dasypyrum* comprises two well-differentiated haplotypes. *Aegilops speltoides* and the polyploid wheat species form three groups: (1) most *Ae. speltoides* accession form a clade of their own (**S**), (2) some *Ae. speltoides* accessions group together with *Triticum timopheevii*, *T. zhukovskyi* and the artificially synthesized wheat *T. kiharae* (**G**) and (3) all accessions of *T. turgidum* and *T. aestivum* share the same haplotype (**B**). The not supported placement of one *Ae. speltoides* accession (PI_48721) close to the **S** group, shifts to a supported position in the **G** group when regions with an high extent of missing data were masked. Additionally, the usage of entire chloroplast genomes resolves that diploid *Triticum* species (**A**) diverged before the **D**-genome taxa and the remaining *Aegilops* species and *Amblyopyrum*. The phylogeny also indicates that **D’** is closely related but distinct from **D**. Further, **M°**, **T** and **U** taxa form a clade, that diverged before the split of taxa having a **C**, **N**, **M** or S* genome. Within this clade the sister species relationship of *Aegilops comosa* and *Ae. uniaristata* is confirmed. *Aegilops comosa* (**M**) groups distinct from the other **M°** plastid type. The species *Ae. searsii*, *Ae. bicornis*, *Ae. longissima*, *Ae. sharonensis* form a clade together with the polyploid *Ae. kotschyi* and *Ae. peregrina* (S*) indicating only very little sequence variation. Concordant to the *ndh*F tree, one sequence each of *Ae. markgrafii* (AE_1831), *Ae. biuncialis* (KP_2012_119) and *Ae. neglecta* (AE_586) group apart from the other sequences of their respective taxon.

### Ages of clades

Divergence times were estimated based of *trn*K-*mat*K, *rbc*L and *ndh*F sequences for each accession included in the study and using an uncorrelated lognormal clock model and a secondary calibration on the MRCA of *Brachypodium distachyon* and Triticeae in *BEAST. Different ages for the split of Triticeae and *Bromus* were obtained depending on the in- or exclusion of the genus *Psathyrostachys*. Including *Psathyrostachys*, Triticeae are paraphyletic and the ages are slightly older but with larger and overlapping 95% highest-posterior densities (HPD) compared to the dataset that does not comprise *Psathyrostachys* (Additional file 5: Figure S2, Additional file 6: Figure S3). In the analysis including *Psathyrostachys* the most recent common ancestor (MRCA) of Triticeae and *Bromus* occurred approximately 19.44 Ma (95% HPD = 12.66-27.20). The split of *Bromus* and the remaining Triticeae (termed “core Triticeae”) occurred approximately 15.77 Ma (95% HPD = 9.38-22.75). The age of this split does not seem affected by the absence of *Psathyrostachys* (15.41 Ma, 95% HPD = 10.72-20.83). However, the MRCA of the core Triticeae occurred approximately 12.17 Ma (95% HPD = 7.65-17.44) including *Psathyrostachys* and nearly 2.5 million years later (9.68 Ma, 95% HPD = 7.42-12.21) in the analysis omitting this early diverging lineage. The MRCA of *Aegilops*, *Triticum* and *Amblyopyrum* (plus *Taeniatherum*) occurred around 4.14 Ma (95% HPD = 2.48-6.44) including *Psathyrostachys* and 3.38 Ma (95% HPD = 2.35-4.47), when omitting it.

## Discussion

### Plant materials

The analysed accessions were mainly acquired from several seed banks (i.e. ICARDA, IPK, USDA, the Czech Crop Research Institute) but additional material was collected during field trips. Multiple accessions per species and intra-specific entities were selected to be able to detect intraspecific genetic variability.

The performance of genome size measurements allowed the distinction of ploidy level differences for accessions of the same species. Our finding of different ploidy levels within *Agropyron cristatum*, *Eremopyrum bonaepartis*, *Pseudoroegneria strigosa, Aegilops crassa* and *Ae. neglecta* are in agreement with previous work [70–74]. For the first time we report the occurrence of different ploidy levels for *Pseudoroegneria stipifolia*.

Few accessions have been found having unexpected genome sizes, like in *Thinopyrum*. Concerns about the condition of seed bank material have been raised in other studies and are related to the fact that it is often maintained under conditions that permit open pollination over several rounds of seed replication [75, 76]. As Triticeae show species-specific genome sizes [67, 77, 78] the performance of flow cytometric measurements is a good strategy to detect problematic material, especially in the case of perennial Triticeae where inflorescences for morphological species determination cannot always be obtained within the timeframe of a research project. Also in this study, a few selected accessions needed to be excluded due to deviations in genome size or morphological characters. However, the vast majority of the material did not reveal any peculiarities and samples directly collected in the wild always grouped with other samples of the same species.

### Sequence assembly

In this study we assembled the chloroplast *ndh*F gene and complete chloroplast genomes using for the latter off-target sequence reads of a target-enrichment approach and NGS sequencing for a comprehensive set of Triticeae taxa. The *ndh*F gene could be assembled for 194 accessions representing 53 Triticeae and three outgroup species without missing data, as it was included in the bait design for sequence enrichment. We obtained a set of 183 whole chloroplast genome sequences that provide new plastid genomes of 36 Triticeae species out of 15 genera for which so far no such sequence was available. From these data we estimated the maternal relationships within Triticeae. In previous studies off-target reads have been successfully analysed in diverse organism groups [36, 79–82]. Because the chloroplast occurs in high copy number in the cells, it constitutes the main fraction of off-target reads in target-enrichment approaches in plants. Therefore the majority of reads identified as chloroplast DNA originated most probably from this genome and not from parts that were transferred from the chloroplast to the nuclear genome, which should be rare in off-target reads.

The pooling of samples from multiple conspecific individuals allowed us to overcome the low coverage for individual samples and to assemble chloroplast genomes to be used as taxon-specific reference for the assembly of individual chloroplast genomes for accessions for which no conspecific reference was available in GenBank. Stretches of missing data remain in the final individual-based assemblies of the plastid genomes. As these stretches occur randomly along the chromosome, they do not influence the detection of structural differences (indels) between chloroplast genomes of species and/or genera. Generally, indels and base substitutions occur mostly in spacer regions of the Triticeae chloroplast genomes. An increase in sequencing depth may have allowed assembling the chloroplast genomes of all individuals without any missing data. However, the comparison of accessions sequenced with different depths shows that overall higher sequencing coverage will not guarantee a complete chloroplast sequence, as off-target regions are randomly (or not) retained during the enrichment process. The most problematic part in assembling the reads was to reach confidence about the detected indel positions, as the short read length of 2 × 100 bp of the Illumina platform did not always cover such regions completely. The whole genome sequences we provide were carefully checked manually and compared to available sequences in GenBank. Comparable to other studies (e.g. [32, 43]) we were not able to confirm all parts of GenBank-derived sequences obtained from whole-genome shotgun sequencing. It might be that they contain some non-identified assembly errors. With the now available longer Illumina paired-end reads of 2 × 250 bp these problems should become less severe in future studies. Finally, the topologies validated our assembly procedure, as previously published GenBank sequences always grouped in their respective clades irrespective of the small differences found.

### Maternal phylogeny of Triticeae

In this work we aimed for a molecular phylogeny of the chloroplast lineages in Triticeae. The results from *ndh*F and whole chloroplast genome phylogenetic analyses are mainly in agreement with hypotheses previously published for groups within the tribe [9, 26, 83] and with respect to the domesticated wheats and their close relatives [30, 31, 84]. Compared to these latter publications a better understanding was obtained, particularly because of the comprehensive taxon sampling, the usage of whole chloroplast genomes, and the inclusion of multiple individuals per species.

The tribe Triticeae is generally accepted to be monophyletic [22, 23, 85–87] with *Bromus*, the only genus in the tribe Bromeae, being the sistergroup to all Triticeae [88, 89]. However, based on our data, but also previously published chloroplast data [26, 35, 90], the monophyly of Triticeae was either rejected or not supported. As morphology [23] and also phylogenies based on nuclear data place *Psathyrostachys* at the base of Triticeae close to *Hordeum* ([10]; own unpublished data), we see two possibilities to explain the chloroplast phylogeny. Thus, either *Psathyrostachys* obtained the chloroplast of a close and nowadays extinct relative belonging to the ancestral Triticeae-Bromeae gene pool, or vice versa an ancestor belonging to the *Bromus* stem group obtained a chloroplast from early Triticeae. In any case, a chloroplast phylogeny including *Bromus* and *Psathyrostachys* might not reflect Triticeae relationships very well, at least for its basal groups, and will also influence the outcome of molecular dating approaches (see below).

The retrieved chloroplast phylogeny indicates a common maternal ancestor for the genera *Australopyrum*, *Eremopyrum*, *Agropyron* and *Henrardia*, with *Eremopyrum*, *Agropyron* and *Henrardia* currently having overlapping distribution areas in southern Europe and western Asia. The monogenomic genus *Australopyrum* (**W**) and all allopolyploid taxa possessing a **W** genome (*Stenostachys* - **HW**, *Anthosachne* - **StYW**, *Connorochloa* – **StYHW**; taxa not sampled) are endemic to dry and temperate Australasia [91]. This supports speciation in allopatry after long-distance dispersal of an *Australopyrum* progenitor and likely recurrent formation of allopolyploid taxa involving numerous other Triticeae species in Australasia. A sister relationship between the species of *Agropyron* and *Eremopyrum* has also been proposed by other studies. However, when *Eremopyrum bonaepartis* was included, *Eremopyrum* became polyphyletic with the diploid cytotype being sister to *Henrardia*. This is in agreement with earlier findings [10, 92, 93].

Similar to Mason-Gamer [83] we found that *Pseudoroegneria*, *Dasypyrum* and *Thinopyrum* form a monophyletic clade indicating that they belong to the same maternal lineage. A sister relationship of *Pseudoroegneria* and *Dasypyrum* has been proposed recently by Escobar et al. [10] based on nuclear data. In our dataset *Dasypyrum* groups however within *Pseudoroegneria*. Within *Dasypyrum*, accessions from Bulgaria and Italy cluster together, while material from Turkey and Greece form another sub-clade. Hence, this pattern may indicate some recent local differentiation. The polyphyletic grouping of *Thinopyrum* within this clade can be explained either by incomplete lineage sorting (ILS) or because *Thinopyrum* repeatedly captured different plastid types of *Pseudoroegneria.* A close relationship to the *Aegilops*-*Triticum*-*Amblyopyrum* group has been reported for *Thinopyrum* based on nuclear data [3, 83, 93–95]. This incongruence might be explained by the fact that *Thinopyrum*, but also *Dasypyrum* and *Pseudoroegneria* are outcrossing taxa [10, 96], which seems to increase the chance of chloroplast capture via hybridization and back-crossing [25]. Moreover, most taxa have overlapping distribution areas in the Caucasus region, also facilitating hybridization. Our results revealed no major sequence variation among chloroplast genomes of *Secale strictum* and *S. cereale*/*S. vavilovii*. This points to an only recent diversification within this genus.

It is well known that the species of *Triticum*, *Aegilops* and *Amblyopyrum muticum* are closely related and of rather recent origin [7, 10, 20, 26]. To date, there is no general agreement on how taxa within this species complex are related to each other, even at the diploid level. There is an on-going dispute if *Aegilops* and *Triticum* should be merged into one genus, and if *Amblyopyrum muticum* should be included into *Aegilops* [74, 84, 97–99]. In agreement with Bordbar et al. [9], the chloroplast phylogeny revealed that *Am. muticum* possesses a chloroplast genome similar to the **M** and **U** genome groups, although based on nuclear data *Am. muticum* appears to be sister to all *Aegilops* and *Triticum* species [7]. The *Aegilops*-like chloroplast genome of *Am. muticum* might be explained by the existence of a common ancestor and therefore a chloroplast genome already shared before divergence of these lineages. Alternatively, it may indicate that it captured the chloroplast from one of these species or their MRCA, which is geographically possible, as distribution areas overlap in Turkey and Armenia.

Polyploid *Triticum* species and *Aegilops speltoides* formed a clade supporting that *Ae. speltoides* is the maternal donor of polyploid wheat genomes. The relationships within this clade corroborate the hypothesis that two different *Ae. speltoides* lineages were involved in their formation [30, 74, 100, 101]. The direct maternal donor for *Triticum timopheevii* and *T. zhukovskyi* (**G**) could be identified, as they share the chloroplast haplotype of three *Ae. speltoides* accessions originating from Iraq and Syria. However the donor remains uncertain for *Triticum turgidum* and *T. aestivum* (**B**), indicating that either our sampling of *Ae. speltoides* was not sufficient to cover the species diversity or pointing to a nowadays extinct donor lineage. Alternatively, Gornicki et al. [30] suggested, that tetraploidisation within this clade predates the one of *T. timopheevii*.

All taxa of the genus *Triticum* s.str. Fall into one clade together with *Aegilops* and *Amblyopyrum*. *Triticum* taxa that were elevated to species rank by Dorofeev et al. [102] could not be distinguished on the basis of their chloroplast haplotypes, which supports the taxonomic treatment of van Slageren [97] subsuming them under the same species name (Additional file 1: Table S1).

Based on chloroplast data and supported by the findings of Petersen et al. [7] and Li et al. [84], *Ae. speltoides* (**S**) appears to be the species that diverged earliest from all other *Aegilops* species. Generally the wheat group is characterized by short branch lengths and plastid haplotypes shared by multiple species. This is most probably due to the only recent divergence of these species.

### Chloroplast capture as indicator of hybridization events

The exchange of chloroplasts among closely related plant species has been reported in diverse plant groups and the effect of hybridization on Triticeae taxa is a matter of discussion. For example, a homoploid hybrid origin of the **D**-genome lineage involving the **A**- and **B**-genome lineages is the subject of a recent dispute [20, 84, 98, 99]. However, our and previous studies [30, 31, 84] revealed three independent but closely related chloroplast lineages with plastids of the **A**-genome lineage being more closely related to the ones of the **D** genome, which can be explained by consecutive divergence. Hence, if such a hybridization event occurred it only affected the nuclear genome.

Although recent publications agree that the detection of hybridization events depends mainly on taxon sampling [19], so far all postulated hypotheses for Triticeae are based on a limited choice of taxa. In our study, three possible cases of ancient chloroplast captures were identified, i.e. for (1) *Bromus*/*Psathyrostachys*, (2) *Thinopyrum* and (3) *Amblyopyrum*, as the chloroplast phylogeny looks considerably different from phylogenies retrieved from nuclear data [7, 83]. More recent events of chloroplast captures were identified for single accessions of the species *Aegilops biuncialis*, *Ae. markgrafii*, *Ae. neglecta* and *Ae. triuncialis* that grouped within clades of other closely related species. We assume such hybridization events to occur frequently between various taxa of the wheat group due to incomplete reproductive isolation among these young species.

### Ages of clades

To obtain dated phylogenies of Triticeae we used the split of *Brachypodium* and Triticeae as secondary calibration point [20] based on *trn*K-*mat*K, *rbc*L and *ndh*F sequences. Pros and cons of using chloroplast data for the estimation of divergence times were already discussed by Middleton et al. [31] who argued that splits of chloroplast lineages might be older than the respective species, resulting in overestimated taxon ages for medium-aged and young clades. For dating in Triticeae we see an additional concern using chloroplast data. Due to mostly low substitution rates in plastid genomes [103] also underestimation of ages is possible in young clades, as fixation of mutations occur as a stochastic process [30, 104, 105] that might be slower than species diversification. In these cases already well-diverged taxa might still possess very similar or identical chloroplast haplotypes [106], resulting in lower age estimations in comparison to nuclear data. This might be the case for many nodes of our tree, although the divergence times retrieved for the main splits are generally about 1 million years older than the ones obtained by Middleton et al. [31]. Our analyses suggest the occurrence of a MRCA for the *Aegilops*/*Triticum* group at approximately 4 Ma, while divergence times of this complex were proposed to date back to approximately 3 Ma [31] or 6.55 Ma based on a dataset of five nuclear and one plastid gene [20].

Another critical topic regarding chloroplast-based dating in Triticeae results from the chloroplast data of *Psathyrostachys*. Our results support the hypothesis that the chloroplast of either *P. juncea* or a *Bromus* ancestor was obtained through chloroplast capture from a taxon belonging the *Bromus*/Triticeae stem lineage, resulting in *P. juncea* clearly falling outside the otherwise monophyletic Triticeae. We strongly favour an event of chloroplast capture over ILS as the cause for the observed relationships. The pronounced sequence variation between *Bromus*, *Psathyrostachys* and the remaining Triticeae for entire chloroplast genomes is best explained by strong and independent sequence divergence of *Bromus* and *Psathyrostachys* compared to the remaining Triticeae. Moreover, in case ILS represents the reason for the observed relationships our coalescent-analyses should have returned the same age for the MRCA of Triticeae-Bromeae with and without the inclusion of *Psathyrostachys*. However, we obtained age estimations that differed by approximately 4 million years.

As the direction of chloroplast capture remains unknown, we estimate the MRCA of all Triticeae to an age of between 10 and 19 million years. When comparing in- vs. exclusion of *P. juncea* the age estimations for all clades are robust, as they fall generally within the 95% HPD (Additional file: 5: Figure S2, Additional file 6: Figure S3).

## Conclusions

We assembled chloroplast sequence data of a large set of monogenomic Triticeae and polyploid wheats by combining on- as well as off-target reads of a sequence-capture approach coupled with Illumina sequencing. This approach allowed us to produce a set of 183 Triticeae chloroplast genomes. These sequences provide new plastid genomes for 39 Triticeae, two *Bromus* and one *Brachypodium* species. Moreover, the data was used to estimate the most comprehensive hypothesis of relationships among Triticeae chloroplast lineages to date.

We infer that an early event of chloroplast capture was involved in the evolution of *Psathyrostachys* or *Bromus*. Either *Psathyrostachys* or *Bromus* obtained a chloroplast from a taxon closely related to a common ancestor of the Triticeae-Bromeae lineage that diverged approximately 19.44 Ma, as the *Psathyrostachys* chloroplast haplotype groups at a deeper node than *Bromus* in our whole-genome phylogeny. We can, however, not safely determine the direction of chloroplast exchange in this case, as this would need the inclusion of much more Bromeae species.

We identified taxa that share the same maternal lineage (e.g. *Agropyron*, *Eremopyrum* and *Heteranthelium*; *Pseudoroegneria* and *Dasypyrum*). Conflicts to nuclear phylogenies (i.e. the grouping of *Thinopyrum*, *Amblyopyrum*) likely indicate old events of chloroplast introgression, while some cases of pronounced intraspecific variation could be attributed to recent events of hybridization, as foreign chloroplast types grouped within otherwise monophyletic species groups (i.e. *Ae. biuncialis* and *Ae. markgrafii*, *Ae. neglecta*).

As plastids are maternally inherited in these grasses, they provide supplementary information to nuclear data. For example, the plastid data indicate the polyphyly of *Eremopyrum*. Moreover, the possession of an *Aegilops*-like chloroplast type of *Amblyopyrum* might reject a taxonomic treatment completely separate from *Aegilops*. Hence, plastid data can facilitate understanding Triticeae evolution, which in turn is crucial on the way to a robust taxonomic system for the entire tribe of Triticeae. However, plastid phylogenies will never be able to infer all hybridization events involved in speciation, e.g. when nuclear genomes got introgressed while chloroplast lineages remains unaffected.

## Additional files

Additional file 1: Table S1. Accessions considered in the study. Overview of the material considered in this study. For all materials, the GenBank identifier, the accession and species name as used in this study (Species) as well as their species synonyms used in the donor seed banks or in the NCBI GenBank (Material source/Reference) are provided. The genome symbol, and the country of origin, where the material was originally collected are given. The ploidy level measured in the scope of this study and the information if a herbarium voucher could be deposited in the herbarium of IPK Gatersleben (GAT) is given. Genomic formulas of tetraploids and hexploids are given as “female x male parent”. The genomes of *Aegilops* taxa follow Kilian et al. [74] and Li et al. [84]. Genome denominations for *Hordeum* follow Blattner [107] and Bernhardt [12] for the remaining taxa. (XLS 84 kb)

Additional file 2: Table S2. Read numbers mapping to the complete chloroplast sequences and *ndh*F. Number of reads mapping and mean coverage for the entire chloroplast genome and *ndh*F after the removal of duplicated reads. Also the proportions of all reads mapping to the chloroplast that mapped to *ndh*F are given. (XLS 66 kb)

Additional file 3: Table S3. Marginal likelihoods and Bayes factor evaluation of Triticeae chloroplast relationships. Stepping-stone estimates of marginal likelihoods calculated with MrBayes 3.2.6 on the *ndh*F dataset and Bayes factor estimated as 2(H_1_-H_2_), where H_1_ enforces monophyly and H_2_ enforces polyphyly of Triticeae chloroplasts. BF_12_ < −10 indicates strong support for model 2. (DOC 27 kb)

Additional file 4: Figure S1. Full representation of the Bayesian phylogenetic tree based on whole chloroplast genome sequences. The multiple sequence alignment comprised 183 genomes assembled in the present study and 39 genomes that were downloaded from GenBank. *Brachypodium distachyon* was used as outgroup taxon. The tree shown is based on the complete alignment of 123,531 base pairs (bp). Posterior probabilities (pp) for the main clades are depicted next to the nodes if they were higher then 0.75. Support values of a second Bayesian analysis based on 114,788 bp of whole chloroplast genomes were alignment positions with more than 50% of missing data were masked are shown below the values of the corresponding nodes in the complete chloroplast analysis if the values differed between analyses. For clades comprising multiple taxa, the taxon affiliation of single accession is indicated by the same symbols behind accession and taxon name (e.g. ‘;“, *). The ploidy level is provided in brackets after the taxon label. Single accessions grouping apart from other accessions of their taxon are shown in bold. To the right the genomic groups are indicated. The red circle represents the secondary calibration point from Marcussen et al. [20] used for node calibrations in multispecies coalescent analyses (MSC). Major nodes are shown in blue. Their estimated ages in million years are given in the box. Two age values for the same node correspond to the analysis with *Psathyrostachys* (first value) and without it (second value). For more information on the results of the MSC analyses see Additional file 5: Figure S2 and Additional file 6: Figure S3. For the full representation of the tree showing the grouping of all single accessions see Additional file 4: Figure S1. For species synonyms see Additional file 1: Table S1. Arrows with support values indicate the nodes they refer to. (PDF 555 kb)

Additional file 5: Figure S2. Calibrated species trees based on *trn*K-*mat*K, *rbc*L, and *ndh*F including *Psathyrostachys*. Calibrated multispecies coalescent derived from three chloroplast loci t*rn*K-*mat*K, *rbc*L and *ndh*F of all Triticeae accessions (excluding polyploid wheats). Sequences of *Brachypodium distachyon*, *Oryza sativa* and *Zea mays* were included as outgroups. Posterior probability values are given for all nodes. Divergence time estimates were inferred using the secondary calibration points from Marcussen et al. [20] for the *Brachypodium*-Triticeae split (mean 44.44 million years ago). Node bars indicate the age range with 95% interval of the highest probability density. For the analysis *Triticum monococcum* and *T. boeoticum*, *Secale cereale* and *S. vavilovii*, *Pseudoroegneria tauri* and *Ps*. *libanotica*, *Taeniatherum caput-medusae* and *Tae*. *crinitum*, *Agropyron cristatum* and *Agr. cimmericum* were each subsumed under a single species name (Additional file 1: Table S1). (JPEG 1085 kb)

Additional file 6: Figure S3. Calibrated species trees based on *trn*K-*mat*K, *rbc*L, and *ndh*F omitting *Psathyrostachys*. Calibrated multispecies coalescent derived from three chloroplast loci t*rn*K-*mat*K, *rbc*L and *ndh*F considering all genomic Triticeae groups covered in the study but omitting *Psathyrostachys* and polyploid wheats. Sequences of *Brachypodium distachyon*, *Oryza sativa* and *Zea mays* were included as outgroups. Posterior probability values are given for all nodes. Divergence time estimates were inferred using the secondary calibration points from Marcussen et al. [20] for the *Brachypodium*-Triticeae split (mean 44.44 million years ago). Node bars indicate the age range with 95% interval of the highest probability density. For the analysis *Triticum monococcum* and *T. boeoticum*, *Secale cereale* and *S. vavilovii*, *Pseudoroegneria tauri* and *Ps*. *libanotica*, *Taeniatherum caput-medusae* and *Tae*. *crinitum*, *Agropyron cristatum* and *Agr. cimmericum* were each subsumed under a single species name (Additional file 1: Table S1). (JPEG 1082 kb)
