# Supplementary material for: Dated tribe-wide whole chloroplast genome phylogeny indicates recurrent hybridizations within Triticeae
Source: BMC Evol Biol. 2017 Jun 16;17:141. doi: 10.1186/s12862-017-0989-9 (PMC5474006; doi:10.1186/s12862-017-0989-9)
Supplement: Supplementary file 3 — Marginal likelihoods and Bayes factor evaluation of Triticeae chloroplast relationships. Stepping-stone estimates of marginal likelihoods calculated with MrBayes 3.2.6 on the ndhF dataset and Bayes factor estimated as 2(H1-H2), where H1 enforces monophyly and H2 enforces polyphyly of Triticeae chloroplasts. BF12 < −10 indicates strong support for model 2. (DOC 27 kb) [file 12862_2017_989_MOESM3_ESM.doc]

**Table S3 Stepping-stone estimates of marginal likelihoods calculated with MrBayes 3.2.6 on the *ndh*F dataset and Bayes factor estimated as 2(H_1_-H_2_), where H_1_ enforces monophyly and H_2_ enforces polyphyly of Triticeae chloroplasts. BF_12_ <-10 indicates strong support for model 2.**

| Topology | Monophyly (1) | Polyphyly (2) |
| --- | --- | --- |
| Mean marginal likelihood (ln) | -6703.6 | -6685.42 |
| Standard deviation | 8.54 | 3.14 |
| BF_12_ | -36.36 | |
