# Supplementary figures and images for: Dated tribe-wide whole chloroplast genome phylogeny indicates recurrent hybridizations within Triticeae
Source: BMC Evol Biol. 2017 Jun 16;17:141. doi: 10.1186/s12862-017-0989-9 (PMC5474006; doi:10.1186/s12862-017-0989-9)

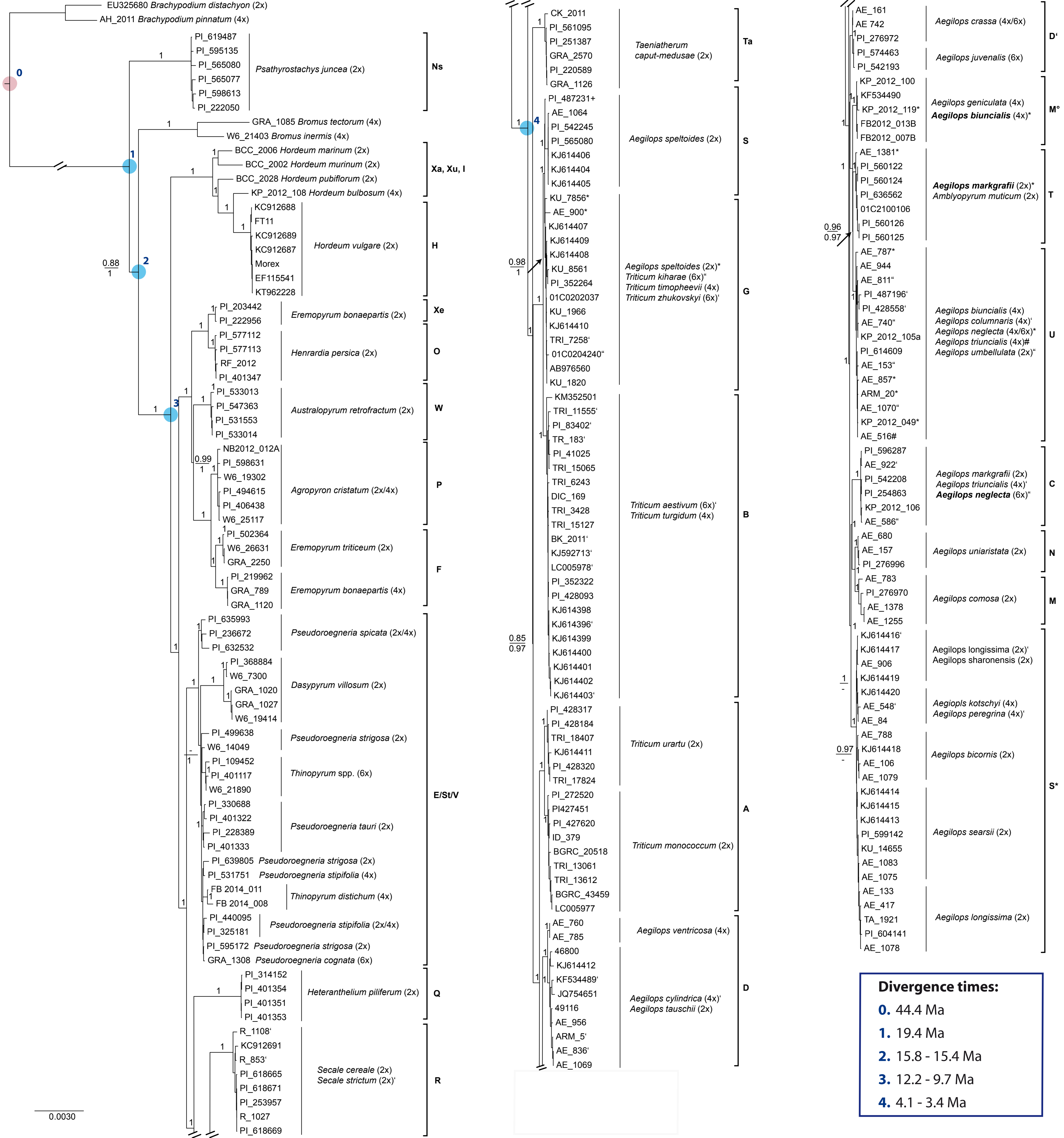

Supplement: Supplementary file 4 — Full representation of the Bayesian phylogenetic tree based on whole chloroplast genome sequences. The multiple sequence alignment comprised 183 genomes assembled in the present study and 39 genomes that were downloaded from GenBank. Brachypodium distachyon was used as outgroup taxon. The tree shown is based on the complete alignment of 123,531 base pairs (bp). Posterior probabilities (pp) for the main clades are depicted next to the nodes if they were higher then 0.75. Support values of a second Bayesian analysis based on 114,788 bp of whole chloroplast genomes were alignment positions with more than 50% of missing data were masked are shown below the values of the corresponding nodes in the complete chloroplast analysis if the values differed between analyses. For clades comprising multiple taxa, the taxon affiliation of single accession is indicated by the same symbols behind accession and taxon name (e.g. ‘;“, *). The ploidy level is provided in brackets after the taxon label. Single accessions grouping apart from other accessions of their taxon are shown in bold. To the right the genomic groups are indicated. The red circle represents the secondary calibration point from Marcussen et al. [20] used for node calibrations in multispecies coalescent analyses (MSC). Major nodes are shown in blue. Their estimated ages in million years are given in the box. Two age values for the same node correspond to the analysis with Psathyrostachys (first value) and without it (second value). For more information on the results of the MSC analyses see Additional file 5: Figure S2 and Additional file 6: Figure S3. For the full representation of the tree showing the grouping of all single accessions see Additional file 4: Figure S1. For species synonyms see Additional file 1: Table S1. Arrows with support values indicate the nodes they refer to. (PDF 555 kb) [file 12862_2017_989_MOESM4_ESM.pdf]

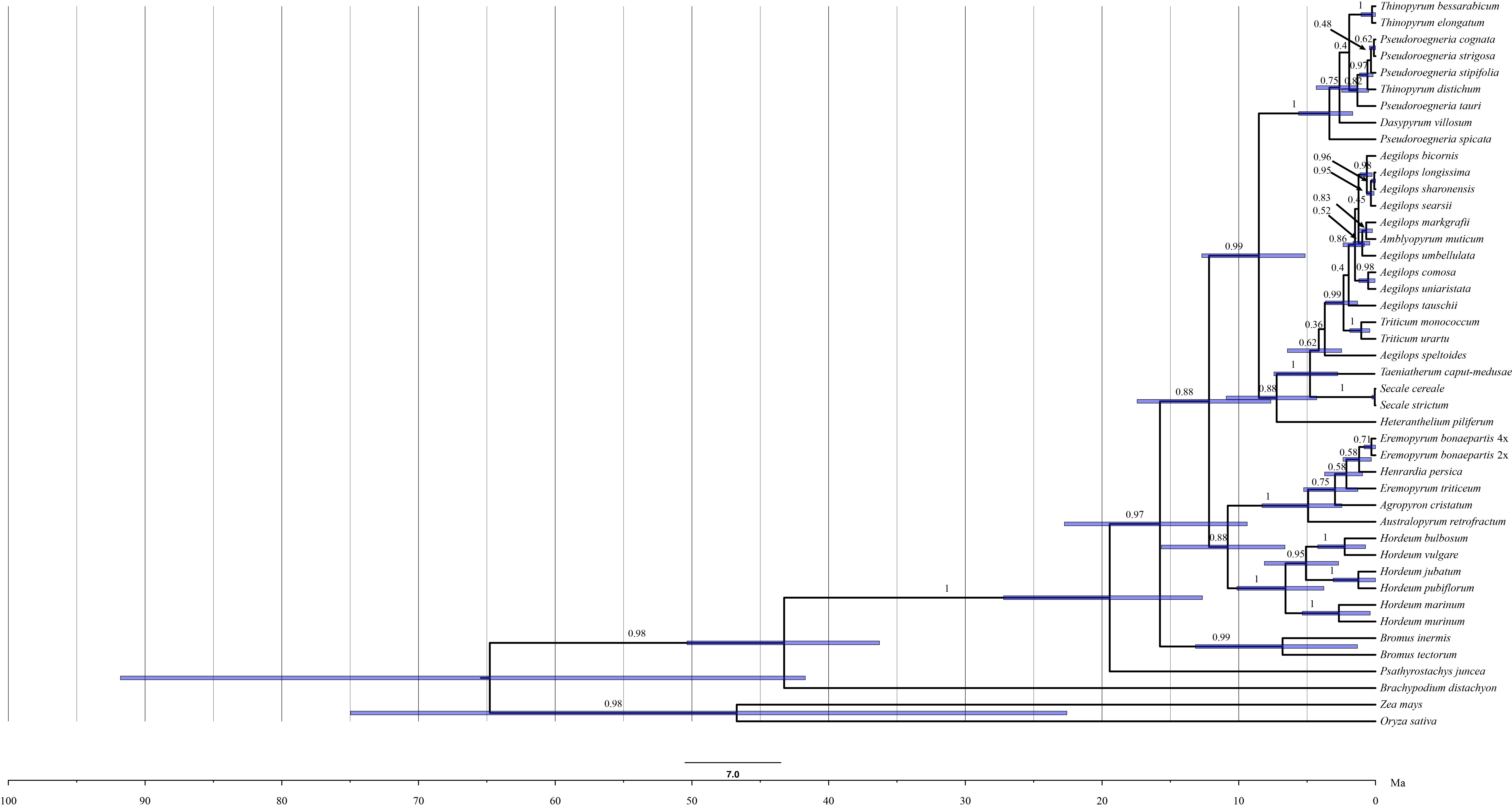

Supplement: Supplementary file 5 — Calibrated species trees based on trnK-matK, rbcL, and ndhF including Psathyrostachys. Calibrated multispecies coalescent derived from three chloroplast loci trnK-matK, rbcL and ndhF of all Triticeae accessions (excluding polyploid wheats). Sequences of Brachypodium distachyon, Oryza sativa and Zea mays were included as outgroups. Posterior probability values are given for all nodes. Divergence time estimates were inferred using the secondary calibration points from Marcussen et al. [20] for the Brachypodium-Triticeae split (mean 44.44 million years ago). Node bars indicate the age range with 95% interval of the highest probability density. For the analysis Triticum monococcum and T. boeoticum, Secale cereale and S. vavilovii, Pseudoroegneria tauri and Ps. libanotica, Taeniatherum caput-medusae and Tae. crinitum, Agropyron cristatum and Agr. cimmericum were each subsumed under a single species name (Additional file 1: Table S1). (JPEG 1085 kb) [file 12862_2017_989_MOESM5_ESM.jpg]

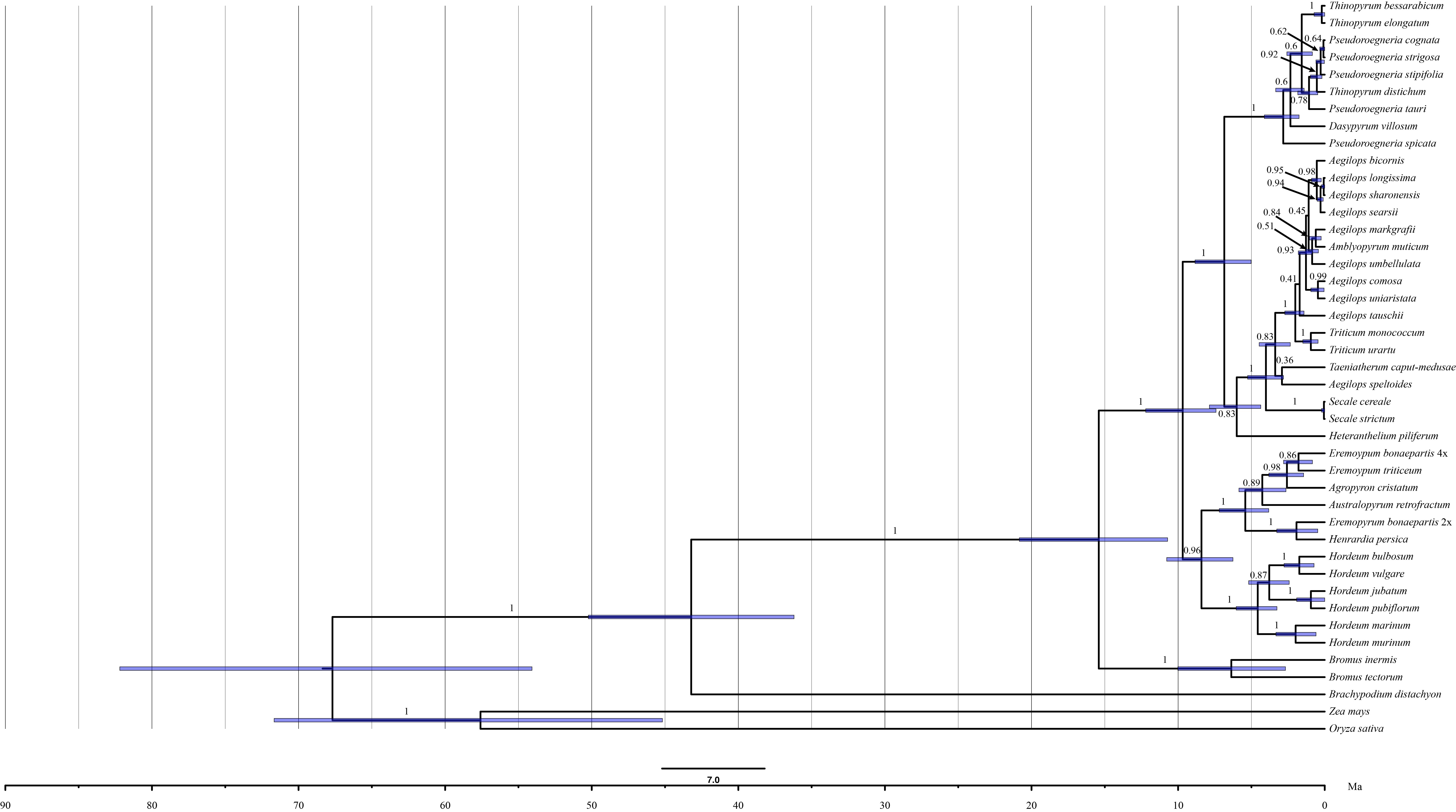

Supplement: Supplementary file 6 — Calibrated species trees based on trnK-matK, rbcL, and ndhF omitting Psathyrostachys. Calibrated multispecies coalescent derived from three chloroplast loci trnK-matK, rbcL and ndhF considering all genomic Triticeae groups covered in the study but omitting Psathyrostachys and polyploid wheats. Sequences of Brachypodium distachyon, Oryza sativa and Zea mays were included as outgroups. Posterior probability values are given for all nodes. Divergence time estimates were inferred using the secondary calibration points from Marcussen et al. [20] for the Brachypodium-Triticeae split (mean 44.44 million years ago). Node bars indicate the age range with 95% interval of the highest probability density. For the analysis Triticum monococcum and T. boeoticum, Secale cereale and S. vavilovii, Pseudoroegneria tauri and Ps. libanotica, Taeniatherum caput-medusae and Tae. crinitum, Agropyron cristatum and Agr. cimmericum were each subsumed under a single species name (Additional file 1: Table S1). (JPEG 1082 kb) [file 12862_2017_989_MOESM6_ESM.jpg]
